# The weight of work: the association between maternal employment and overweight in low- and middle-income countries

**DOI:** 10.1186/s12966-017-0522-y

**Published:** 2017-10-18

**Authors:** Vanessa M. Oddo, Sara N. Bleich, Keshia M. Pollack, Pamela J. Surkan, Noel T. Mueller, Jessica C. Jones-Smith

**Affiliations:** 1 Department of International Health, Center for Human Nutrition, Johns Hopkins Bloomberg School of Public Health, 615 N. Wolfe St, Baltimore, MD 21205 USA; 2 Department of Health Policy and Management, Harvard T.H. Chan School of Public Health, 677 Huntington Avenue, Boston, MA 02115 USA; 3 Department of Health Policy and Management, Johns Hopkins Bloomberg School of Public Health, 624 N. Broadway, Baltimore, MD 21205 USA; 4 Department of International Health, Social and Behavioral Interventions Program, Johns Hopkins Bloomberg School of Public Health, 615 N. Wolfe St, Baltimore, MD 21205 USA; 5 Department of Epidemiology, Johns Hopkins Bloomberg School of Public Health, 615 N. Wolfe St, Baltimore, MD 21205 USA; 6 Nutrition Sciences Program & Department of Health Services, University of Washington School of Public Health, Raitt Hall, Seattle, WA 98195 USA

**Keywords:** Maternal employment, Overweight, Low- and middle-income countries, Nutrition transition

## Abstract

**Background:**

Maternal employment has increased in low-and middle-income countries (LMIC) and is a hypothesized risk factor for maternal overweight due to increased income and behavioral changes related to time allocation. However, few studies have investigated this relationship in LMIC.

**Methods:**

Using cross-sectional samples from Demographic and Health Surveys, we investigated the association between maternal employment and overweight (body mass index [BMI] ≥ 25 kg/m^2^) among women in 38 LMIC (*N* = 162,768). We categorized mothers as formally employed, informally employed, or non-employed based on 4 indicators: employment status in the last 12 months; aggregate occupation category (skilled, unskilled); type of earnings (cash only, cash and in-kind, in-kind only, unpaid); and seasonality of employment (all year, seasonal/occasional employment). Formally employed women were largely employed year-round in skilled occupations and earned a wage (e.g. professional), whereas informally employed women were often irregularly employed in unskilled occupations and in some cases, were paid in-kind (e.g. domestic work). For within-country analyses, we used adjusted logistic regression models and included an interaction term to assess heterogeneity in the association by maternal education level. We then used meta-analysis and meta-regression to explore differences in the associations pooled across countries.

**Results:**

Compared to non-employed mothers, formally employed mothers had higher odds of overweight (pooled odds ratio [POR] = 1.3; 95% Confidence Interval [CI] 1.2, 1.4) whereas informally employed mothers, compared to non-employed mothers, had lower odds of overweight (POR = 0.72; 95% CI: 0.64, 0.81). In 14 LMIC, the association varied by education. In these countries, the magnitude of the formal employment-overweight association was larger for women with low education (POR = 1.5; 95% CI: 1.1, 1.9) compared to those with high education (POR = 1.2; 95% CI: 1.0, 1.3).

**Conclusions:**

Formally employed mothers in LMIC have higher odds of overweight and the association varies by educational attainment in 14 countries. This knowledge highlights the importance of workplace initiatives to reduce the risk of overweight among working women in LMIC.

**Electronic supplementary material:**

The online version of this article (doi:10.1186/s12966-017-0522-y) contains supplementary material, which is available to authorized users.

## Background

The launch of the Millennium Development Goals (MDG) raised global interest, awareness, and attention to women’s employment as several MDG targets identified promoting women’s employment as an intervention strategy to improve health and alleviate poverty. Subsequently, over the past two decades, there have been vast increases in employment and shifts from part-time to full-time work among women in low- and middle-income countries (LMIC) [1, 2]. These increases are paralleled by increases in the prevalence of overweight and a changing nutritional landscape, characterized by shifts in diet (e.g. increases in consumption of processed foods) and physical activity (e.g. decreases in energy expenditure) [3–9]. Higher rates of maternal employment may provide greater exposure to these changes of modernity and adversely impact bodyweight. As environments change, a better understanding of the relationship between maternal employment and overweight in LMIC is critical given that overweight further threatens the health and economic stability of LMIC and the possible need for additional policies and programs designed to support women in the workplace [10].

Only two prior studies have begun to examine the association between maternal employment and overweight in LMIC. Goryakin and Suhrcke found that that being employed, compared to non-employed, was associated with a higher probability of women being overweight in middle-income countries, but a lower probability of being overweight in low-income countries [11]. A second study found that mothers working in professional occupations had higher odds of overweight compared to those working in agriculture [2]. Key limitations of these studies include using a dichotomous indicator of employment and only reporting regional-level trends.

The primary aim of this study is to address key gaps in the literature about the maternal employment-overweight relationship by using the most recent data available from 38 LMIC to explore country-specific estimates, with mothers categorized as formally employed, informally employed, or non-employed. This unique feature of this investigation allowed for a thorough characterization of employment in LMIC settings, and for a novel investigation of the relationship between maternal employment and maternal overweight. The secondary aims of this paper are to assess heterogeneity in the maternal employment-overweight relationship by maternal education level and countries’ stage in the nutrition transition, as indicated by gross domestic product (GDP) and urbanization. We hypothesize that maternal employment is associated with higher odds of overweight through several mechanisms, including increased income and behavioral changes related to time allocation.

## Methods

### Data source and study population

Data were obtained from the Demographic and Health Surveys (DHS), which are cross-sectional household surveys administered in LMIC. DHS employ a multistage cluster sample design and are nationally representative [12]. The surveys are standardized allowing for cross-country comparisons.

This study specifically focused on mothers, rather than all women more broadly, based on several factors. First, we aimed to keep our sample consistent to prior related studies and earlier years of the DHS only collected anthropometrics among women with children under 5 years old. Second, our a priori hypothesis was that mothers’ time would be more constrained than that of women without children.

Our analyses utilized one survey per country. We retained surveys from 38 LMIC that met the following criteria for inclusion: 1) it was the most recent survey administered from 2010 to the time of analysis (May 2016), 2) women were queried on their employment status and 3) at least 85% of the women surveyed had anthropometric data collected. Our final analytic sample (*n* = 162,768) included non-pregnant women, aged 18 to 49 years, who had children between ages 0–5 years since most DHS surveys collected anthropometrics only among this subgroup. Women whose children resided outside the household surveyed were also excluded (*n* = 173). The Johns Hopkins Institutional Review Board deemed that this analysis of de-identified secondary data was not human subjects research.

### Primary dependent variable

Overweight or obese, defined as body mass index (BMI) ≥ 25 kg/m^2^ (henceforth referred to as overweight), served as our primary dependent variable [13]. Height (in centimeters [cm] to the nearest 0.1 cm) and weight (in kilograms [kg] to the nearest 0.1 kg) were measured by trained technicians using standard techniques [14, 15].

### Independent variable

Maternal employment was modeled as a 3-category variable: formally employed, informally employed, and non-employed. Informal employment refers to activities (e.g. domestic work, street vendors, homestead agriculture) and income that are outside regulation and may help woman capitalize on desirable non-wage features of the informal sector, such as flexible hours [16–18]. We separately categorized formal and informal employment based on research indicating that: 1) 60% of women employed in LMIC are engaged in informal employment, 2) factors driving labor force participation differ across labor market sectors, 3) wages are 60–77% lower in the informal sector and 4) the employment-overweight relationship and dietary patterns may vary by the number of hours women work and occupation type [2, 11, 16, 19–24]. Notably, this investigation categorized women as formally employed, informally employed or non-employed, rather than focusing specifically on occupation type, based on qualitative work which suggested that occupation type alone may not capture both the changes in income and time allocation that occur with employment [25]. Additionally, prior literature has focused on the relationship between occupation type (i.e. professional versus agriculture-based occupations) and overweight risk in LMIC [2].

Employment status was defined based on 4 indicators guided by the International Labor Organization’s definitions of formal and informal employment [16, 26]: 1) employment status in the last 12 months (employed, non-employed); 2) aggregate occupation category (skilled, unskilled); 3) type of earnings (cash only, cash and in-kind, in-kind only, unpaid); and 4) seasonality of employment (all year, seasonal/occasional employment). Formal employment included three combinations: 1) employed, skilled occupation, cash only earnings, employed all year; 2) employed, skilled occupation, cash only earnings, seasonal/occasional employment; and 3) employed, unskilled occupation, cash only earnings, employed all year. All other employed women were categorized as informally employed (e.g. employed, unskilled occupation, cash only earnings, seasonal/occasional employment) (see Additional file 1: Table S1).

### Confounders and effect measure modifiers

We identified confounding factors a priori using a directed acyclic graph, which is a causal diagram used to characterize the relationship between the exposure and outcome based on theorized relationships and relationships documented in the literature [27–29]. Confounders included maternal age (years), marital status (married or living together versus single, widowed, divorced), parity, number of household members, child age (months) and childcare support. Living with ones’ mother, mother-in-law, or sister served a proxy for childcare support (a binary variable).

We tested for heterogeneity in the employment-overweight association by maternal education level because prior literature suggests that the employment-nutritional status relationship may vary by educational attainment [30]. Research also shows that women with higher educational attainment more often participate in formal sector employment [16, 19]. We modeled education as a binary variable (< primary school completed versus ≥ primary school completed) based on the distribution of educational attainment across countries.

We additionally explored differences in the country-level associations by log-GDP per capita, adjusted for purchasing power parity (or the financial ability to buy products and services with a sum of money) and percent urban, both theorized drivers of the nutrition transition [8, 31, 32]. GDP-per capita was log transformed due to convention and to reflect the expected influence of a percent increase (e.g. 10%), rather than an absolute dollar increase (e.g. $10 U.S. dollars). We utilized log-GDP and percent urban from the World Development Indicators database, corresponding to the year the survey was administered [33, 34]. Percent urban was defined as the number of people living in urban areas divided by the total population.

### Statistical analysis

We used separate multivariable logistic regression models for each country to test the association between maternal employment and overweight. We utilized sampling weights provided by the DHS, which account for differential probability of selection and response. Analyses additionally accounted for the clustered design through the use of Taylor series linearized standard errors. In our primary models, we tested for heterogeneity in the association between maternal employment and overweight by maternal education level using a post-hoc Wald test and retained the interaction term only if statistically significant. If the association between employment and overweight was homogenous by maternal education level, then education was included in the model as a confounder. Because we retained interaction terms in select countries (*n* = 14), we estimated pooled odds ratios (POR), using random effects meta-analysis, separately for the associations between formal and informal employment and overweight for three subgroups: women in countries where the association did not vary education, women with low educational attainment (< primary) in countries where the association varied by education, and women with high educational attainment (≥ primary) in countries where the association varied by education. Random effects meta-analysis, used to generate pooled odds ratios is the statistical combination of the estimates from separate countries (i.e. it utilizes the country-specific beta coefficient) and assumes that the magnitude of the association between employment and overweight may differ by country. We then assessed differences in the association between countries by log-GDP and urbanization using random-effects meta-regression, which utilized the country-specific effect estimates to statistically test for differences in the employment-overweight effect size by country-level factors (e.g. log-GDP) [35–37]. Alpha was set to 0.05 for main effects and to 0.10 for interaction terms. Analyses were performed using Stata 14.1 (StataCorp LP, College Station, Texas).

As sensitivity analyses, we modeled underweight (BMI < 18.5) and normal weight (BMI ≥ 18.5 to BMI < 25) as outcomes to assess whether the results were consistent with the outcome of overweight. We assessed the robustness of our results when modeling obesity (BMI ≥ 30) and BMI (measured continuously) as outcomes and when controlling for within country residence (urban/rural status). We re-ran our primary models across the full sample of women, which included mothers with children older than 5 years and women without children. Finally, we estimated the difference in the probability of being overweight between formally employed versus non-employed mothers and between informally employed versus non-employed mothers.

## Results

Data were analyzed for 162,768 women from 38 LMIC. Selected sample characteristics by country are detailed in Table 1 and by employment type in Additional file 1: Table S2. The percent of mothers formally (3.7% to 54%) and informally (2.1% to 80%) employed was wide-ranging. Approximately 29% of women were overweight, but prevalence rates across countries were wide-ranging (4.3% to 80%).

**Table 1:** Descriptive characteristics of mothers participating in selected Demographic and Health Surveys

			N (%)^a^			
Country	Year	N^b^	Formally Employed (*n* = 40,723)^b,c^	Informally Employed (*n* = 46,970)^b,c^	≥ Primary Education (*n* = 81,477)^b^	Overweight (*n* = 48,421)^b,d^
Bangladesh	2011	6224	564 (9.0%)	130 (2.1%)	3929 (63%)	776 (12%)
Benin	2011–12	6372	2658 (42%)	1738 (28%)	999 (16%)	1672 (27%)
Burkina Faso	2010	4204	659 (16%)	2802 (66%)	348 (8.2%)	381 (9.0%)
Cambodia	2014	3477	1527 (43%)	1165 (33%)	1578 (44%)	610 (17%)
Cameroon	2011	2965	962 (32%)	1301 (44%)	1530 (51%)	928 (31%)
Colombia^e^	2010	11,809	4071 (38%)	2734 (25%)	9386 (87%)	4832 (45%)
Comoros	2012	1468	341 (23%)	286 (19%)	584 (39%)	703 (47%)
Cote d’Ivoire	2011–12	2017	544 (28%)	900 (47%)	355 (19%)	444 (23%)
DRC	2013–14	4293	697 (16%)	2753 (65%)	2014 (48%)	638 (15%)
Dominican Republic	2013	2385	1147 (48%)	227 (10%)	1846 (78%)	1202 (51%)
Egypt	2014	9765	1090 (11%)	238 (2.4%)	7517 (77%)	7762 (80%)
Ethiopia	2011	6095	874 (14%)	2659 (42%)	434 (6.8%)	276 (4.3%)
Ghana	2014	1760	807 (47%)	612 (36%)	998 (59%)	703 (41%)
Guinea	2012	1978	470 (24%)	1120 (57%)	239 (12%)	347 (18%)
Haiti	2012	2879	879 (32%)	845 (30%)	1259 (45%)	734 (26%)
Honduras	2011–12	7368	2262 (32%)	1219 (17%)	4786 (68%)	3684 (53%)
Kyrgyz Republic^f^	2012	2686	559 (22%)	86 (3.4%)	2261 (89%)	858 (34%)
Lesotho	2014	1034	248 (25%)	153 (15%)	814 (81%)	463 (46%)
Liberia	2013	2110	562 (30%)	610 (32%)	640 (34%)	464 (24%)
Malawi	2010	3026	508 (17%)	1750 (57%)	789 (26%)	502 (16%)
Mali	2012–13	2308	301 (13%)	658 (28%)	220 (9.4%)	382 (16%)
Mozambique	2011	5848	601 (10%)	2296 (38%)	1157 (19%)	812 (13%)
Namibia	2013	1376	440 (34%)	126 (10%)	1012 (79%)	416 (32%)
Nepal	2011	1681	182 (11%)	1073 (62%)	738 (43%)	173 (10%)
Niger	2012	2771	316 (11%)	413 (15%)	172 (6.1%)	504 (18%)
Nigeria	2013	15,052	8243 (54%)	2598 (17%)	7288 (48%)	3772 (25%)
Pakistan	2012–13	2106	292 (14%)	303 (14%)	815 (38%)	711 (33%)
Peru^g^	2012	7202	3267 (49%)	1341 (20%)	5542 (82%)	4041 (60%)
Rwanda	2010	2721	277 (10%)	2210 (80%)	649 (24%)	439 (16%)
Sierra Leone	2013	3194	583 (18%)	2071 (64%)	706 (22%)	520 (16%)
Tajikistan	2012	2957	334 (11%)	496 (16%)	2902 (95%)	845 (28%)
Tanzania	2010	4269	750 (17%)	3103 (71%)	2772 (63%)	821 (19%)
Timor-Leste	2009–10	4724	317 (6.7%)	1478 (31%)	2431 (51%)	281 (5.9%)
Togo	2013–14	2073	1046 (53%)	633 (32%)	638 (32%)	559 (28%)
Uganda	2011	1187	372 (32%)	540 (47%)	419 (36%)	203 (18%)
Yemen	2013	8476	312 (3.7%)	512 (6.1%)	3961 (48%)	2217 (27%)
Zambia	2013–14	7520	1414 (19%)	2915 (39%)	4001 (53%)	1652 (22%)
Zimbabwe	2010–11	3388	522 (15%)	876 (26%)	2931 (87%)	1003 (30%)
Total	162,768		40,998 (25%)	46,970 (29%)	80,659 (50%)	47,330 (29%)

*DRC* Democratic Republic of Congo

^a^Number of observations (percentage) were estimated using the country sample weight

^b^Total sample size in each country and by subgroup (e.g. total formally employed) are unweighted

^c^Type of employment was based on 4 following indicators: employment during the last 12 months (yes, no); aggregate occupation category (skilled, unskilled); type of earnings (cash only, cash and in-kind, in-kind only, unpaid); and seasonality of employment (all year, seasonally/occasionally)

^d^Overweight was defined as BMI ≥ 25 kg/m^2^

^e^Employment type was based on employment status, occupation category and earnings only because seasonality of employment was not queried in this survey

^f^Maternal education level was dichotomized as < secondary level of education complete and ≥ secondary level of education complete

^g^Employment type was based on employment status, type of earnings, and seasonality only because occupation type was not queried in this survey

Being formally employed, compared to non-employed, was associated with 30% higher odds of maternal overweight when pooling estimates across 38 LMIC (POR = 1.3; 95% Confidence Interval [CI] 1.2, 1.4) (Table 2). On the contrary, informal employment was associated with lower odds of being overweight in 18 LMIC and when pooling estimates across all countries (POR = 0.72; 95% CI: 0.64, 0.81).

**Table 2:** Pooled odds ratio for the relationship between formal and informal maternal employment and overweight^a^

	*N* = 162,768^b^
	Pooled Odds Ratio (95% Confidence Interval)
Formal Employment^c^	
All low- and middle-income countries	1.3 (1.2, 1.4)
Countries where the association did not vary by education	1.2 (1.1, 1.4)
Countries where the association varied by education: low education^d^	1.5 (1.1, 1.9)
Countries where the association varied by education: high education^d^	1.2 (1.0, 1.3)
Informal Employment^c^	
All low- and middle-income countries	0.72 (0.64, 0.81)
Countries where the association did not vary by education	0.71 (0.60, 0.83)
Countries where the association varied by education: low education^d^	0.74 (0.62, 0.88)
Countries where the association varied by education: high education^d^	0.63 (0.50, 0.79)

^a^Pooled odds ratios (POR) were generated using meta-analysis and pool estimates across country subgroups. Overweight was defined as BMI ≥ 25 kg/m^2^. All models were adjusted for maternal age (years), parity, marital status (married, not married), number of household members, child age (months), and substitute childcare provider (yes, no). Models which did not retain the employmentXeducation interaction term were also adjusted for maternal education (< primary education, ≥ primary education completed)

^b^Total sample size is unweighted

^c^Type of employment was based on 4 indicators: 1) employment during the last 12 months (yes, no); 2) aggregate occupation category (skilled, unskilled); 3) type of earnings (cash only, cash and in-kind, in-kind only, unpaid); and 4) seasonality of employment (all year, seasonally/occasionally)

^d^The employmentXeducation interaction term was retained in the following countries: Bangladesh, Benin, Burkina Faso, Democratic Republic of Congo, Ethiopia, Honduras, Kyrgyz Republic, Mozambique, Nigeria, Peru, Rwanda, Tanzania, Uganda, Zimbabwe. The relative difference in the employment-overweight association, comparing mothers with high to low education for formal employment was: POR = 0.78 (95% CI: 0.62, 0.98) and for informal employment was: POR = 0.81 (95% CI: 0.58, 1.1)

### Heterogeneity in the employment-overweight association by maternal education

**Fig. 1:**
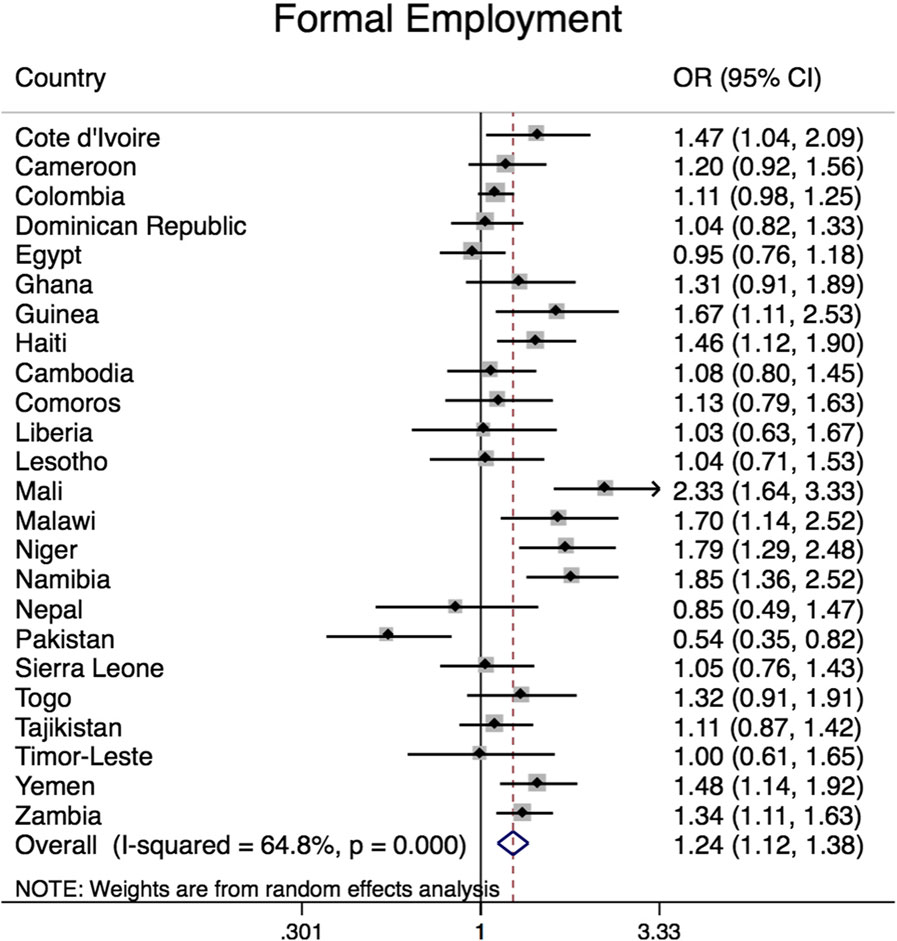
Adjusted logistic regression for the relationship between maternal employment and overweight, in countries where the association did not vary by education^1, 2^. ^1^Country-specific odds ratios were estimated using logistic regression to test the relationship between employment and overweight (BMI ≥ 25 kg/m^2^). Overall odds ratios were generated using meta-analysis and pool estimates across countries.^2^ Models were adjusted for maternal age (years), parity, marital status (married, not married), number of household members, child age (months), substitute childcare provider (yes, no) and maternal education (< primary education, ≥ primary education)

Figure 1 details the country-specific relationships between formal and informal employment and overweight in the 24 countries where the association did not vary by maternal level of education. Formally employed mothers, compared to non-employed mothers, had higher odds of overweight in 10 of 24 LMIC (Fig. 1). Informally employed mothers, compared to non-employed mothers, had lower odds of overweight in 13 of 24 LMIC (Fig. 1). When pooling estimates across countries where the association did not vary by maternal education, formally employed women had higher odds of overweight compared to those who were non-employed (POR = 1.2; 95% CI: 1.1, 1.4) (Table 2). In countries where the association did not vary by education, informally employed women had lower odds of overweight compared to non-employed women (POR = 0.71; 95% CI: 0.60, 0.83).

**Fig. 2:**
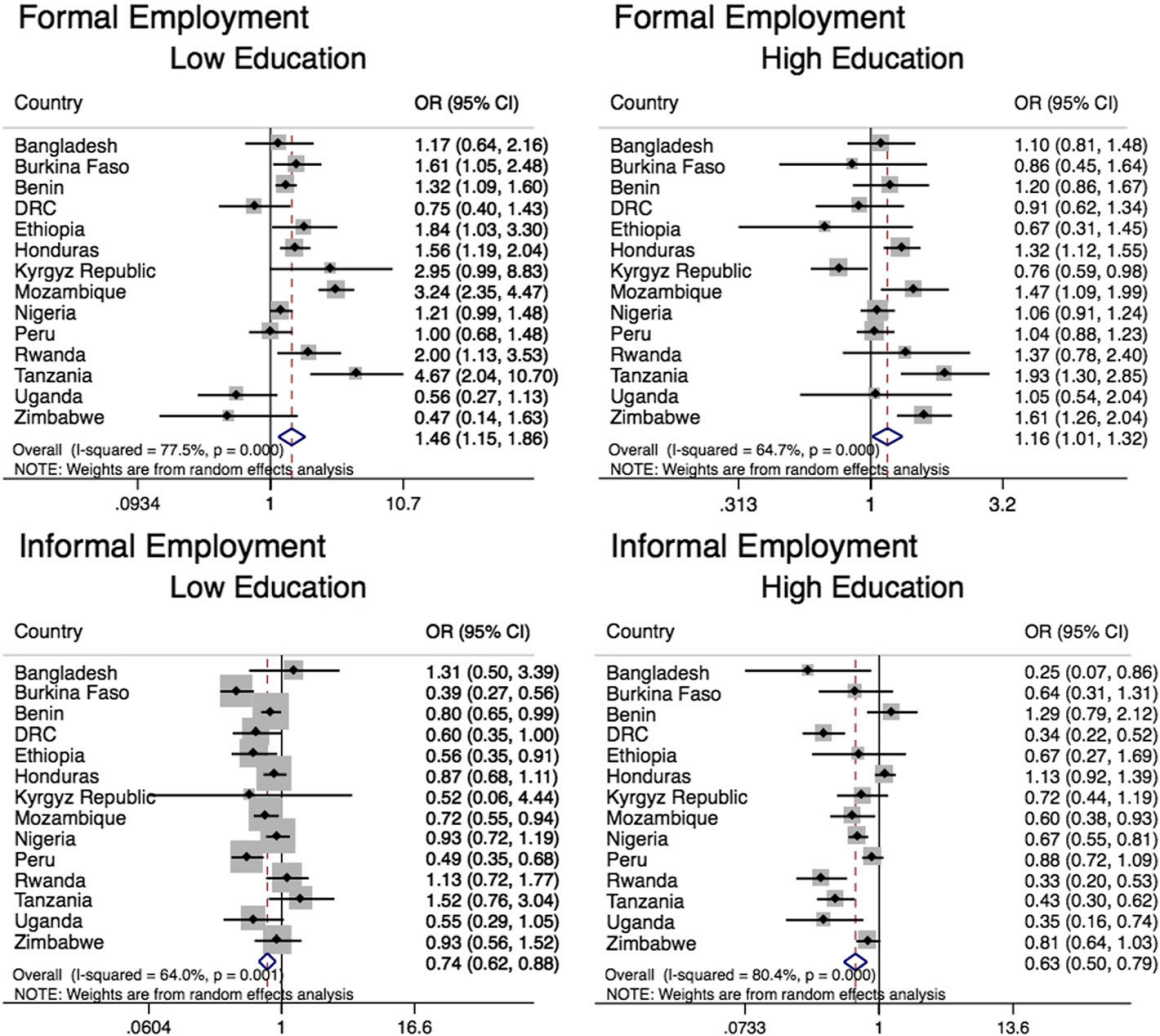
Adjusted logistic regression for the relationship between maternal employment and overweight, in countries where the association varied by education^1, 2^. DRC = Democratic Republic of Congo.^1^ Country-specific odds ratios were estimated using logistic regression to test the relationship employment and overweight (BMI ≥ 25 kg/m^2^). Overall odds ratios were generated using meta-analysis and pool estimates across countries.^2^ All models were adjusted for maternal age (years), parity, marital status (married, not married), number of household members, child age (months), and substitute childcare provider (yes, no). Models included an employmentXeducation interaction term (< primary education, ≥ primary education completed)

In 14 LMIC, the association between employment and overweight varied by maternal education level (Fig. 2 and Table 2). In countries where the association varied by education, the formal employment-overweight association was larger for women with low education (POR = 1.5; 95% CI: 1.1, 1.9) as compared to the association among those with high education (POR = 1.2; 95% CI: 1.0, 1.3) (POR for the difference [high vs. low] = 0.78; 95% CI: 0.62, 0.98) (Table 2). However, the association was positive in both cases. Both informally employed women with low education (POR = 0.74; 95% CI: 0.62, 0.88) and high education (POR = 0.63; 95% CI: 0.50, 0.79) had lower odds of overweight compared to non-employed women. But the magnitude of the employment-overweight association was not different for informally employed women with high education as compared to the analogous association among informally employed women with low education (POR for the difference [high vs. low] = 0.81; 95% CI: 0.58, 1.1) (Table 2).

### Meta-regression

The meta-regression results indicated that neither the formal employment-, nor informal employment-overweight associations were statistically significantly different by log-GDP or urbanization (Additional file 1: Table S3, Figure. S1 and S2).

### Sensitivity analyses

Consistent with our results for overweight, formal employment was associated with lower odds of underweight (POR = 0.75; 95% CI: 0.67, 0.83) (Additional file 1: Table S4) and lower odds of normal weight (POR = 0.89; 95% CI: 0.83, 0.95). Informal employment was not associated with underweight (POR = 0.99; 95% CI: 0.89, 1.1), but results indicated that informal employment was associated with higher odds of normal weight (POR = 1.2; 95% CI: 1.1, 1.3).

The direction of the country-specific and pooled associations between both formal and informal employment were robust to modeling obesity as the outcome (Additional file 1: Table S5) and modeling continuous BMI as the outcome (Additional file 1: Table S6). The associations between employment and overweight were also similar in direction and magnitude for the full sample of women (formal POR = 1.4; 95% CI: 1.3, 1.5; informal POR = 0.76; 95% CI: 0.68, 0.85) (Additional file 1: Table S7) and when controlling for within country residence (urban versus rural) (formal POR = 1.2; 95% CI: 1.1, 1.3; informal POR = 0.80; 95% CI: 0.72, 0.88) (Additional file 1: Table S8). Consistent with our primary results, formally employed mothers, compared to non-employed mothers, have a higher probability of being overweight (predicted probability: 0.043; 95% CI: 0.026, 0.060) (Additional file 1: Table S9 and Figure. S3 and S4). On the contrary, but also consistent with our primary models, informally employed mothers have a lower probability of being overweight compared to non-employed mothers (predicted probability: -0.045; 95% CI: -0.064, −0.026).

## Discussion

This study utilizes nationally representative data from 38 LMIC to investigate the within- and between-country associations between maternal employment and overweight. It is important to better understand the benefits, as well as the potential unintended consequences, of maternal employment on weight status in order to inform intervention approaches that both support women’s participation in the labor force and address increasing overweight prevalence in LMIC. Formally employed mothers had higher odds of overweight, while informally employed mothers had lower odds of overweight. The employment-overweight association varied by maternal education level in several countries. The magnitude of the association between employment and overweight was smaller among formally employed mothers with higher education, compared to lower education. Our results also suggested that the employment-overweight relationship did not vary by GDP or urbanization.

Our results for formal employment are largely consistent with previous studies from middle-income countries and high-income countries (where formal employment is predominant), which find that maternal employment is associated with higher odds of overweight [2, 22–24]. Formal employment could result in a positive energy balance and subsequently, higher odds of overweight, through several mechanisms. Increased economic resources, stemming from maternal employment, are associated with changes in food procurement behaviors, including increasing household food expenditures and increasing the purchase of energy-dense foods as they become more affordable, which could lead to an overconsumption of energy [38, 39]. Evidence finds an association between labor force participation and consumption of alcohol and meat in LMIC [4]. Behavioral changes related to mother’s time allocation may also play a role. Data from the United States indicate that women working full-time spend less time cooking [40]. This is consistent with evidence from LMIC which suggests increases in work hours is associated with significantly lower energy intakes from home and higher intakes from commercially prepared foods [41]. Labor-related physical activity may also decrease as jobs emerging in the formal labor sector are more sedentary and shifting patterns of employment from agriculture into the service sector are associated with overweight among women in LMIC [42]. Maternal mental health may also play a role. Dual parental and employment responsibilities may induce stress among mothers and modest evidence suggests that adverse mental health (e.g. depression) is associated with increased overweight among women [43–46]. It is possible that the rapid transformation of the food and built environment in LMIC, such as the spread of supermarkets and availability of ultra-processed foods, provides an enabling environment for the aforementioned hypothesized changes among formally employed women [47–49].

Limited prior evidence, which pools formal and informal employment, suggests that women’s employment is associated with a lower probability of being overweight in low-income countries, where informal employment is predominate [11]. This is consistent with our finding which indicates that informal employment is associated with lower odds of overweight. One explanation for this finding could be that the smaller increases in income and more active occupations associated with informal work may act to prevent women from becoming overweight. Wages in the informal sector employment are 60–77% lower than wages in the formal sector and we hypothesize that more nominal increases in income may illicit different food purchasing behaviors as compared to formally employed women [16]. For example nominal increases in income may allow women to somewhat increase their food expenditures, but not enable them to ‘trade up’ to energy-dense foods [50]. Substantive dietary changes may not occur until income increases beyond 15% of household expenditures; in a predominantly informally employed sample in Guatemala, for example, employed women reported that some higher-fat, animal source foods largely remained unaffordable [25, 51]. The informal sector includes mothers who voluntarily engage in employment due to part-time hours, which may afford women more flexibility in balancing domestic and employment responsibilities, rendering them less reliant on prepared foods with fewer time constraints [16–18]. Informally employed women are also likely to be engaged in occupational physical activity, particularly those employed in agriculture-based occupations.

Our results suggested heterogeneity in the employment-overweight associations by education in some contexts. It is likely that the combination of higher educational-attainment, increased income, and exposure to more prosocial ideas stemming from interactions with educated, skilled co-workers make formally employed mothers (with relatively high-education) more inclined to allocate resources in ways that are less conducive to weight gain, compared to their lower education, formally employed counterparts. These women may have improved decision-making power, increased efficiency of time use, and be somewhat less inclined to purchase energy-dense foods. Another explanation for the employment-overweight association being larger in magnitude among formally employed mothers with lower education derives from the mismatch of early life with later life environment. Mothers with low educational attainment may have had fewer resources early in life, but now these women have ubiquitous access to calories, increasing their propensity for being overweight [52]. Nevertheless, some aspects of formal employment seem to be associated with a positive energy balance among all formally employed mothers, irrespective of education-level.

Currently, there is a gap in knowledge on how to effectively develop and implement obesity-prevention and control strategies in LMIC. However, there is a consensus that multi-sectoral approaches should be implemented and one such component includes behavioral settings such as the workplace. These results suggest that employers in LMIC should consider implementing workplace strategies that promote healthy eating and physical activity and reduce overweight. For example, employers could consider offering individual-level nutrition education, in combination with environmental approaches to spur behavior change, such as increasing the availability of healthier food and beverage choices in the workplace.

Our study is not without limitations. Due to our study design, we cannot establish temporality or rule out unmeasured or residual confounding. The generalizability of our findings may be limited to women who have children under 5 years of age; however, the results were largely unchanged when we examined women with older children. Moreover, our results can only be generalized to countries that DHS fielded from 2010 to 2016. Our proxy measure of childcare support, which was included as a confounder, was limited in that we do not know if the potential substitute childcare provider was also employed. Finally, evidence suggested that there are two segments of the informal employment sector (voluntary and forced), but we were unable to distinguish between these sectors using the DHS [16].

Despite these limitations, this study has two key strengths which improve upon the existing evidence: 1) we estimate both within- and between-country associations among 38 countries and 2) we distinguish non-employed women from formally and informally employed women.

## Conclusions

This study enhances our understanding of the association between maternal employment and overweight in LMIC. We provide evidence that mothers engaged in the formal workforce in LMIC have higher odds of overweight and that the employment-overweight association varies by educational attainment in several contexts. A better understanding of this area is critical given the expected increases in women’s labor force participation in LMIC in coming decades. This new knowledge may also be helpful for the development of policies and programs to reduce the risk of overweight among working women in LMIC. Future research exploring the mechanisms of the employment-overweight association and the workplace will help inform potential environmental and contextual interventions (e.g. food offerings at work, transportation to work, and programs to decrease sedentary activity at work) that may mitigate this association.

## Additional file

Additional file 1: Additional material includes supplemental tables and figures to support the main manuscript. (DOCX 7866 kb)

## References

[1] Adair L, Guilkey D, Bisgrove E, Gultiano S (2002). Effect of childbearing on Filipino women's work hours and earnings. J Popul Econ.

[2] Lopez-Arana S, Avendano M, van Lenthe FJ, Burdorf A (2013). Trends in overweight among women differ by occupational class: results from 33 low-and middle-income countries in the period 1992–2009. Int J Obes.

[3] NCD Risk Factor Collaboration (2016). Trends in adult body-mass index in 200 countries from 1975 to 2014: a pooled analysis of 1698 population-based measurement studies with 19.2 million participants. Lancet.

[4] Dave D, Doytch N, Kelly IR (2016). Nutrient intake: a cross-national analysis of trends and economic correlates. Soc Sci Med.

[5] Du S, Mroz TA, Zhai F, Popkin BM (2004). Rapid income growth adversely affects diet quality in China—particularly for the poor!. Soc Sci Med.

[6] Wiggins S, Keats S, Han E, Shimokawa S, Hernández J, Claro R. The rising cost of a healthy diet. London: Overseas Development Institute; 2015. https://www.odi.org/sites/odi.org.uk/files/odi-assets/publications-opinion-files/9580.pdf. Accessed 28 May 2016.

[7] Popkin BM (2001). The nutrition transition and obesity in the developing world. J Nutr.

[8] Popkin BM, Du S (2003). Dynamics of the nutrition transition toward the animal foods sector in China and its implications: a worried perspective. J Nutr.

[9] Dodzin S, Vamvakidis A (2004). Trade and industrialization in developing economies. J Dev Econ.

[10] Raymond SU, Leeder S, Greenberg HM (2006). Obesity and cardiovascular disease in developing countries: a growing problem and an economic threat. Curr Opin Clin Nutr Metab Care.

[11] Goryakin Y, Suhrcke M (2014). Economic development, urbanization, technological change and overweight: what do we learn from 244 demographic and health surveys?. Econ Hum Biol.

[12] Macro ICF (2008). Demographic and health surveys: DHS model questionnaires.

[13] Nishida C, Uauy R, Kumanyika S, Shetty P (2004). The joint WHO/FAO expert consultation on diet, nutrition and the prevention of chronic diseases: process, product and policy implications. Public Health Nutr.

[14] ICF Macro. Training Field Staff for DHS Surveys. Calverton; 2009.

[15] Gibson RS (2005). Principles of nutritional assessment.

[16] Günther I, Launov A (2012). Informal employment in developing countries: opportunity or last resort?. J Dev Econ.

[17] Magnac T. Segmented or competitive labor markets. Econometrica. 1991:165–87.

[18] Pratap S, Quintin E (2006). Are labor markets segmented in developing countries? A semiparametric approach. Eur Econ Rev.

[19] Fields G. A guide to multisector labor market models. 2005. http://siteresources.worldbank.org/SOCIALPROTECTION/Resources/SP-Discussion-papers/Labor-Market-DP/0505.pdf. Accessed 28 May 2016.

[20] Cunningham W, Maloney WF. Heterogeneity among Mexico's micro-enterprises: an application of factor and cluster analysis. World Bank Policy Working Paper. 1999. http://siteresources.worldbank.org/DEC/Resources/Heterogeneity01and0Cluster0Analysis.pdf. Accessed 28 May 2016.

[21] Chen MA (2012). The informal economy: definitions, theories and policies.

[22] Courtemanche C (2008). Working yourself to death? The relationship between work hours and obesity.

[23] Au N, Hauck K, Hollingsworth B (2013). Employment, work hours and weight gain among middle-aged women. Int J Obes.

[24] Au N, Hollingsworth B (2011). Employment patterns and changes in body weight among young women. Prev Med.

[25] Oddo VM, Surkan PJ, Hurley KM, Lowery C, de Ponce S, Jones-Smith JC. Pathways of the Association Between Maternal Employment and Weight Status among Women and Children: Qualitative Findings from Guatemala. Matern Child Nutr. 2017;e12455. 10.1111/mcn.12455. PMC5668210 28464549

[26] Hussmanns R. Measuring the informal economy: From employment in the informal sector to informal employment. Integration Working Paper. 2004. http://www.ilo.org/wcmsp5/groups/public/---dgreports/---integration/documents/publication/wcms_079142.pdf. Accessed 28 May 2016.

[27] Textor J, Hardt J, Knuppel S (2011). DAGitty: a graphical tool for analyzing causal diagrams. Epidemiology.

[28] Greenland S, Pearl J, Robins JM. Causal diagrams for epidemiologic research. Epidemiology. 1999:37–48. 9888278

[29] Pearl J (2003). Causality: models, reasoning and inference. Economic Theory.

[30] Galobardes B, Morabia A, Bernstein MS (2000). The differential effect of education and occupation on body mass and overweight in a sample of working people of the general population. Ann Epidemiol.

[31] Nandi A, Sweet E, Kawachi I, Heymann J, Galea S (2014). Associations between Macrolevel economic factors and weight distributions in low-and middle-income Countries: a multilevel analysis of 200 000 adults in 40 Countries. Am J Public Health.

[32] Ezzati M, Hoorn SV, Lawes CMM, Leach R, James WPT, Lopez AD (2005). Rethinking the" diseases of affluence" paradigm: global patterns of nutritional risks in relation to economic development. PLoS Med.

[33] The World Bank. World Development Indicators: Gross Domestic Product per capita, based on purchasing power parity. 2016 http://data.worldbank.org/indicator/NY.GDP.PCAP.PP.CD. Accessed 28 May 2016.

[34] The World Bank. World Development Indicators: Percent Urban. 2016. http://data.worldbank.org/indicator/SP.URB.TOTL.IN.ZS?view=chart Accessed 28 May 2016.

[35] Baker W, Michael White C, Cappelleri J, Kluger J, Coleman C (2009). Understanding heterogeneity in meta-analysis: the role of meta-regression. Int J Clin Pract.

[36] Thompson SG, Higgins J (2002). How should meta-regression analyses be undertaken and interpreted?. Stat Med.

[37] Benton T (2014). Using meta-regression to explore moderating effects in surveys of international achievement. Pract Assessment Res Eval.

[38] Popkin BM (1980). Time allocation of the mother and child nutrition. Ecol Food Nutr.

[39] Engle PL (1993). Influences of mothers' and fathers' income on children's nutritional status in Guatemala. Soc Sci Med.

[40] Cawley J, Liu F (2012). Maternal employment and childhood obesity: a search for mechanisms in time use data. Econ Hum Biol.

[41] Bisgrove EZ, Popkin BM (1996). Does women's work improve their nutrition: evidence from the urban Philippines. Soc Sci Med.

[42] Goldin C (1995). The U-shaped female labor force function in economic development and economic history. Schultz TP Investment in Women’s human capital and economic Development.

[43] Slater J, Sevenhuysen G, Edginton B, O'Neil J (2011). ‘trying to make it all come together’: structuration and employed mothers' experience of family food provisioning in Canada. Health Promot Int.

[44] de Wit L, Luppino F, van Straten A, Penninx B, Zitman F, Cuijpers P (2010). Depression and obesity: a meta-analysis of community-based studies. Psychiatry Res.

[45] Scott KM, Bruffaerts R, Simon GE, Alonso J, Angermeyer M, de Girolamo G (2008). Obesity and mental disorders in the general population: results from the world mental health surveys. Int J Obes.

[46] Simon GE, Ludman EJ, Linde JA, Operskalski BH, Ichikawa L, Rohde P (2008). Association between obesity and depression in middle-aged women. Gen Hosp Psychiatry.

[47] Reardon T, Berdegue JA (2002). The rapid rise of supermarkets in Latin America: challenges and opportunities for development. Dev Policy Rev.

[48] Reardon T, Timmer CP, Barrett CB, Berdegué J (2003). The rise of supermarkets in Africa, Asia, and Latin America. Am J Agric Econ.

[49] Monteiro CA, Moubarac JC, Cannon G, Ng SW, Popkin B (2013). Ultra-processed products are becoming dominant in the global food system. Obes Rev.

[50] Popkin BM, Du S (2003). Dynamics of the nutrition transition toward the animal foods sector in China and its implications: a worried perspective. J Nutr.

[51] Leroy JL, Ruel M, Verhofstadt E (2009). The impact of conditional cash transfer programmes on child nutrition: a review of evidence using a programme theory framework. J J Dev Effect.

[52] Bateson P, Gluckman P, Hanson M (2014). The biology of developmental plasticity and the predictive adaptive response hypothesis. J Physiol.

