# Supplementary material for: The weight of work: the association between maternal employment and overweight in low- and middle-income countries
Source: Int J Behav Nutr Phys Act. 2017 Oct 18;14:66. doi: 10.1186/s12966-017-0522-y (PMC6389244; doi:10.1186/s12966-017-0522-y)
Supplement: Supplementary file 1 — Additional material includes supplemental tables and figures to support the main manuscript. (DOCX 7866 kb) [file 12966_2017_522_MOESM1_ESM.docx]

| **Table S1. Definitions of maternal employment type^1,2^** | |
| --- | --- |
| **Non-Employed** | 1. Not employed in the last 12 months |
| **Formally**  **Employed** | 1. Employed in the last 12 months, skilled occupation, earns cash only, employed all year 2. Employed in the last 12 months, skilled occupation, earns cash only, seasonal/occasional employment 3. Employed in the last 12 months, unskilled occupation, earns cash only, employed all year |
| **Informally**  **Employed** | 1. Employed in the last 12 months, skilled occupation, earns cash and in-kind, employed all year 2. Employed in the last 12 months, skilled occupation, earns cash and in-kind, seasonal/occasional employment 3. Employed in the last 12 months, unskilled occupation, earns cash only, seasonal/occasional employment 4. Employed in the last 12 months, unskilled occupation, earns cash and in-kind, employed all year 5. Employed in the last 12 months, unskilled occupation, earns cash and in-kind, seasonal/occasional employment 6. Employed in the last 12 months, unskilled occupation, unpaid or in kind, employed all year 7. Employed in the last 12 months, unskilled occupation, unpaid or in kind, seasonal/occasional employment |

^1^ Based on the following 4 indicators: 1) self-reported employment status in the last 12 months (employed, non-employed); 2) aggregate occupation category (skilled, unskilled); 3) type of earnings (cash only, cash and in-kind, in-kind only, unpaid); 4) seasonality of employment (employed all year, seasonal or occasional employment).

^2^ Skilled occupations include: professional, clerical, services, and skilled manual labor. Unskilled occupations include: sales, unskilled manual labor, agriculture, and domestic/housekeeping.

| **Table S2. Descriptive characteristics of mothers participating in selected Demographic and Health Surveys, by employment status** | | | | | | | |
| --- | --- | --- | --- | --- | --- | --- | --- |
|  |  |  | **Mean (standard deviation) or N (%)^1^** | | | |  |
| **Country** | | **Overall**  **(n=162,768)^2,3^** | | **Formally Employed**  **(n=40,723)^2,3^** | **Informally Employed**  **(n=47,664)^2,3^** | **Non-employed**  **(n=74,381)^2,3^** |  |
| Mean Age (years) | | 29 (6.9) | | 30 (6.6) | 30 (7.2) | 28 (6.8) |  |
| Mean Number of Children | | 3.1 (2.0) | | 3.0 (1.9) | 3.5 (2.1) | 3.0 (1.9) |  |
| Mean BMI (kg/m^2^) | | 23 (4.7) | | 24 (4.9) | 22 (3.9) | 24 (5.0) |  |
| Overweight^4^ | | 47,330 (29%) | | 15,547 (38%) | 8,748 (19%) | 23,005 (31%) |  |
| Underweight^5^ | | 15,618 (9.7%) | | 2,539 (6.2%) | 4,973 (11%) | 8,105 (11%) |  |
| Household Wealth Category | | | | | | |  |
| Poorest | | 34,025 (21%) | | 5,006 (12%) | 13,458 (29%) | 15,561 (21%) |  |
| Poorer | | 33,862 (21%) | | 6,595 (16%) | 12,268 (26%) | 14,999 (21%) |  |
| Middle | | 33,409 (21%) | | 7,772 (19%) | 10,430 (22%) | 15,207 (21%) |  |
| Richer | | 31,810 (20%) | | 9,972 (24%) | 7,414 (16%) | 14,424 (20%) |  |
| Richest | | 27,982 (17%) | | 11,656 (28%) | 3,397 (7%) | 12,929 (18%) |  |
| Educational Attainment^6^ | | | | | | |  |
| Low Education | | 80,428 (50%) | | 15,536 (38%) | 31,079 (66%) | 33,812 (46%) |  |
| High Education | | 80,661 (50%) | | 25,463 (62%) | 15,889 (34%) | 39,308 (54%) |  |

BMI = body mass index

^1^ Mean (standard deviation) and number of observations (percentage) were estimated using the country-specific sample weight.

^2^ Total sample size overall and sample size in each subgroup are unweighted.

^3^ Type of employment was based on 4 indicators: 1) employment during the last 12 months (yes, no); 2) aggregate occupation category (skilled, unskilled); 3) type of earnings (cash only, cash and in-kind, in-kind only, unpaid); and 4) seasonality of employment (all year, seasonal/occasional).

^4^ Overweight was defined as BMI ≥ 25 kg/m^2^.

^5^ Underweight was defined as BMI < 18.5 kg/m^2^.

^6^ Low education was defined as < primary education complete and high education defined as ≥ primary education complete.

| **Table S3. Meta-regression results for the association between formal and informal maternal employment and overweight by log-GDP and percent urban^1^** | | | | |
| --- | --- | --- | --- | --- |
|  | **β (95% Confidence Interval)** | | | |
|  | **Log-GDP^2^** | | | **Percent Urban^2,3^** |
| Formal Employment^4^ | | |  |  |
| All low- and middle-income countries | | | -0.13  (-0.28, 0.020) | -0.0035  (-0.011, 0.0042) |
| Countries where the association did not vary by education | | | -0.11  (-0.25, 0.034) | -0.0017  (-0.0092, 0.0058) |
| Countries where the association varied by education: low education^5^ | | | -0.098  (-0.55, 0.36) | -0.0067  (-0.028, 0.015) |
| Countries where the association varied by education: high education^5^ | | | -0.051  (-0.26, 0.16) | -0.0016  (-0.012, 0.0087) |
| Informal Employment^4^ |  | | |  |
| All low- and middle-income countries | | 0.025  (-0.13, 0.18) | | 0.012  (-0.34, 0.36) |
| Countries where the association did not vary by education | | 0.035  (-0.16, 0.23) | | 0.0086  (-0.00055, 0.018) |
| Countries where the association varied by education: low education^5^ | | 0.012  (-0.28, 0.30) | | -0.0022  (-0.016, 0.011) |
| Countries where the association varied by education: high education^5^ | | 0.27  (-0.47, 0.58) | | 0.015  (-0.00012, 0.030) |

Log-GDP = log-gross domestic product, per capita

^1^ Beta coefficients were estimated using linear meta-regression. All models were adjusted for maternal age (years), parity, marital status (married, not married), number of household members, child age (months), and substitute childcare provider (yes, no). Models which did not retain the employmentXeducation interaction term were also adjusted for maternal education (< primary education complete, ≥ primary education complete).

^2^ Data were obtained from the World Development Indicators database and correspond to the survey year used.

^3^ Percent urban was defined as the number of people living in urban areas divided by the total population.

^4^ Type of employment was based on 4 indicators: 1) employment during the last 12 months (yes, no); 2) aggregate occupation category (skilled, unskilled); 3) type of earnings (cash only, cash and in-kind, in-kind only, unpaid); and 4) seasonality of employment (all year, seasonal/occasional).

^5^ The employmentXeducation interaction term was retained in the following countries: Bangladesh, Benin, Burkina Faso, Democratic Republic of Congo, Ethiopia, Honduras, Kyrgyz Republic, Mozambique, Nigeria, Peru, Rwanda, Tanzania, Uganda, Zimbabwe.

| **Table S4. Adjusted logistic regression for the relationship between formal and informal maternal employment and underweight^1,2^** | | | | | |
| --- | --- | --- | --- | --- | --- |
|  | |  |  | **Odds Ratio (95% Confidence Interval)** | |
| **Country** | **Year** | **N** | **Formal**  **Employment^3^** | **Informal Employment^3^** |  |
| Bangladesh^4^ | | 2011 | 6,224 | 1.1 (0.75, 1.5) | 0.95 (0.53, 1.7) |
| Benin^4^ | | 2011-2012 | 6,372 | 0.76 (0.56, 1.0) | 0.67 (0.47, 0.96) |
| Burkina Faso^4^ | | 2010 | 4,204 | 0.65 (0.43, 0.96) | 0.83 (0.61, 1.1) |
| Cambodia | | 2014 | 3,477 | 0.94 (0.66, 1.3) | 1.5 (1.1, 2.1) |
| Cameroon | | 2011 | 2,965 | 0.33 (0.21, 0.54) | 0.43 (0.30, 0.62) |
| Colombia^5^ | | 2010 | 11,809 | 0.73 (0.54, 0.98) | 0.67 (0.49, 0.91) |
| Comoros | | 2012 | 1,468 | 0.62 (0.27, 1.4) | 0.94 (0.39, 2.3) |
| Cote d'Ivoire | | 2011-2012 | 2,017 | 1.0 (0.46, 2.2) | 1.2 (0.55, 2.4) |
| DRC^4^ | | 2013-2014 | 4,293 | 0.90 (0.44, 1.9) | 0.99 (0.64, 1.5) |
| Dominican Republic | | 2013 | 2,385 | 0.79 (0.46, 1.3) | 1.5 (0.53, 4.5) |
| Egypt | | 2014 | 9,558 | 2.5 (0.76, 8.5) | --- ^6^ |
| Ethiopia^4^ | | 2011 | 6,095 | 0.99 (0.70, 1.4) | 1.1 (0.88, 1.3) |
| Ghana | | 2014 | 1,760 | 0.54 (0.28, 1.1) | 0.85 (0.44, 1.6) |
| Guinea | | 2012 | 1,978 | 0.61 (0.37, 1.0) | 0.83 (0.54, 1.3) |
| Haiti | | 2012 | 2,879 | 0.77 (0.52, 1.1) | 1.0 (0.70, 1.5) |
| Honduras^4^ | | 2011-2012 | 7,368 | 0.54 (0.25, 1.2) | 0.90 (0.50, 1.6) |
| Kyrgyz Republic^4,7^ | | 2012 | 2,686 | 0.45 (0.047, 4.2) | 1.2 (0.46, 3.0) |
| Lesotho | | 2014 | 1,034 | 0.65 (0.19, 2.2) | 1.3 (0.48, 3.5) |
| Liberia | | 2013 | 2,110 | 0.87 (0.42, 1.8) | 1.3 (0.75, 2.2) |
| Malawi | | 2010 | 3,026 | 0.75 (0.40, 1.4) | 0.86 (0.59, 1.3) |
| Mali | | 2012-2013 | 2,308 | 0.54 (0.32, 0.90) | 0.71 (0.50, 1.0) |
| Mozambique^4^ | | 2011 | 5,848 | 0.55 (0.25, 1.2) | 1.5 (1.1, 2.1) |
| Namibia | | 2013 | 1,376 | 0.58 (0.35, 0.97) | 0.67 (0.35, 1.3) |
| Nepal | | 2011 | 1,681 | 0.54 (0.26, 1.1) | 0.90 (0.62, 1.3) |
| Niger | | 2012 | 2,771 | 0.74 (0.48, 1.2) | 0.87 (0.59, 1.3) |
| Nigeria^4^ | | 2013 | 15,052 | 0.86 (0.71, 1.0) | 0.80 (0.64, 1.0) |
| Pakistan | | 2012-2013 | 2,106 | 1.9 (1.2, 3.1) | 2.9 (1.9, 4.4) |
| Peru^4,8,9^ | | 2012 | 7,028 | 0.35 (0.068, 1.8) | 1.0 (0.25, 3.9) |
| Rwanda^4^ | | 2010 | 2,721 | 0.88 (0.31, 2.5) | 0.86 (0.44, 1.7) |
| Sierra Leone | | 2013 | 3,194 | 0.66 (0.36, 1.2) | 0.95 (0.61, 1.5) |
| Tajikistan | | 2012 | 2,957 | 0.78 (0.47, 1.3) | 1.4 (0.96, 2.2) |
| Tanzania^4^ | | 2010 | 4,269 | 0.081 (0.019, 0.34) | 0.77 (0.45, 1.3) |
| Timor-Leste | | 2009-2010 | 4,724 | 0.82 (0.51, 1.3) | 1.3 (1.1, 1.6) |
| Togo | | 2013-2014 | 2,073 | 0.66 (0.36, 1.2) | 1.1 (0.63, 2.1) |
| Uganda^4^ | | 2011 | 1,187 | 1.1 (0.53, 2.4) | 1.2 (0.59, 2.4) |
| Yemen | | 2013 | 8,476 | 0.67 (0.42, 1.1) | 0.99 (0.77, 1.3) |
| Zambia | | 2013-2014 | 7,520 | 0.63 (0.47, 0.86) | 1.2 (0.98, 1.5) |
| Zimbabwe^4^ | | 2010-2011 | 3,388 | 0.33 (0.047, 2.4) | 0.78 (0.32, 1.9) |
| **Total N/**  **Pooled Odds Ratio** | |  | **162,387** | **0.75 (0.67, 0.83)** | **0.99 (0.89, 1.1)** |

DRC = Democratic Republic of Congo

^1^ Country-specific odds ratios were estimated using logistic regression. Underweight was defined as BMI <18.5 kg/m^2^. Country-specific sample sizes were unweighted. Pooled odds ratios were generated using meta-analysis.

^2^ All models were adjusted for maternal age (years), parity, marital status (married, not married), number of household members, child age (months), and substitute childcare provider (yes, no). Models which did not retain the employmentXeducation interaction term were also adjusted for maternal education (< primary education complete, ≥ primary education complete).

^3^ Type of employment was based on 4 indicators: 1) employment during the last 12 months (yes, no); 2) aggregate occupation category (skilled, unskilled); 3) type of earnings (cash only, cash and in-kind, in-kind only, unpaid); and 4) seasonality of employment (all year, seasonal/occasional).

^4^ Included employmentXeducation interaction term.

^5^ Employment type was based on employment status, occupation and earnings only because seasonality of employment was not queried in this survey.

^6^ Small cell sizes for informally employed women who are underweight prevented us from generating estimates for this subgroup in Egypt (missing values for informally employed n=207).

^7^ Maternal education level was dichotomized as < secondary level of education complete and ≥ secondary level of education complete.

^8^ Employment type was based on employment status, type of earnings, and seasonality only because occupation type was not queried in this survey.

^9^ Missing values (n=174) for availability of substitute childcare provider.

| **Table S5. Adjusted logistic regression for the relationship between formal and informal maternal employment and obesity^1,2^** | | | | | |
| --- | --- | --- | --- | --- | --- |
|  | |  |  | **Odds Ratio (95% Confidence Interval)** | |
| **Country** | **Year** | **N** | **Formal**  **Employment^3^** | **Informal Employment^3^** |  |
| Bangladesh^4^ | | 2011 | 6,224 | 0.83 (0.10, 6.7) | 1.0 (0.12, 8.0) |
| Benin^4^ | | 2011-2012 | 6,372 | 1.6 (1.1, 2.4) | 0.54 (0.34, 0.84) |
| Burkina Faso^4^ | | 2010 | 4,204 | 2.3 (0.96, 5.6) | 0.27 (0.11, 0.63) |
| Cambodia | | 2014 | 3,477 | 0.78 (0.34, 1.8) | 0.41 (0.17, 0.99) |
| Cameroon | | 2011 | 2,965 | 1.1 (0.78, 1.6) | 0.54 (0.36, 0.82) |
| Colombia^5^ | | 2010 | 11,809 | 1.0 (0.86, 1.2) | 1.1 (0.89, 1.3) |
| Comoros | | 2012 | 1,468 | 1.3 (0.82, 2.0) | 1.4 (0.90, 2.3) |
| Cote d'Ivoire | | 2011-2012 | 2,017 | 1.9 (0.97, 3.7) | 1.1 (0.53, 2.3) |
| DRC^4^ | | 2013-2014 | 4,293 | 0.19 (0.020, 1.8) | 0.91 (0.28, 2.9) |
| Dominican Republic | | 2013 | 2,385 | 1.1 (0.81, 1.4) | 1.0 (0.65, 1.7) |
| Egypt | | 2014 | 9,765 | 0.85 (0.73, 1.0) | 0.89 (0.65, 1.2) |
| Ethiopia^4^ | | 2011 | 6,095 | 3.5 (1.1, 11) | 0.56 (0.22, 1.5) |
| Ghana | | 2014 | 1,760 | 0.91 (0.52, 1.6) | 0.61 (0.31, 1.2) |
| Guinea | | 2012 | 1,978 | 2.0 (0.94, 4.1) | 0.61 (0.27, 1.4) |
| Haiti | | 2012 | 2,879 | 1.5 (0.98, 2.3) | 1.1 (0.70, 1.8) |
| Honduras^4^ | | 2011-2012 | 7,368 | 1.3 (0.92, 1.9) | 0.80 (0.58, 1.1) |
| Kyrgyz Republic^4,6^ | | 2012 | 2,686 | 3.1 (0.82, 12) | 0.56 (0.055, 5.7) |
| Lesotho | | 2014 | 1,034 | 1.2 (0.75, 1.9) | 1.1 (0.61, 1.9) |
| Liberia | | 2013 | 2,110 | 2.6 (1.3, 5.2) | 1.7 (0.81, 3.4) |
| Malawi | | 2010 | 3,026 | 2.8 (1.4, 5.6) | 0.64 (0.33, 1.2) |
| Mali | | 2012-2013 | 2,308 | 2.4 (1.5, 4.0) | 0.73 (0.40, 1.3) |
| Mozambique^4^ | | 2011 | 5,848 | 5.9 (3.4, 10) | 0.56 (0.29, 1.1) |
| Namibia | | 2013 | 1,376 | 1.6 (1.0, 2.4) | 0.43 (0.17, 1.1) |
| Nepal | | 2011 | 1,681 | 0.92 (0.40, 2.1) | 0.071 (0.014, 0.35) |
| Niger | | 2012 | 2,771 | 2.2 (1.3, 3.6) | 0.99 (0.56, 1.7) |
| Nigeria^4^ | | 2013 | 15,052 | 1.2 (0.75, 2.1) | 0.85 (0.47, 1.6) |
| Pakistan | | 2012-2013 | 2,106 | 0.57 (0.31, 1.1) | 0.41 (0.20, 0.82) |
| Peru^4,7^ | | 2012 | 7,202 | 1.1 (0.73, 1.8) | 0.38 (0.24, 0.62) |
| Rwanda^4^ | | 2010 | 2,721 | 7.8 (1.7, 35) | 0.78 (0.18, 3.3) |
| Sierra Leone | | 2013 | 3,194 | 1.7 (0.74, 3.8) | 0.51 (0.25, 1.0) |
| Tajikistan | | 2012 | 2,957 | 0.93 (0.55, 1.6) | 0.52 (0.31, 0.85) |
| Tanzania^4^ | | 2010 | 4,269 | 1.8 (0.43, 7.9) | 0.79 (0.23, 2.7) |
| Timor-Leste | | 2009-2010 | 4,724 | 1.4 (0.44, 4.3) | 0.70 (0.28, 1.8) |
| Togo | | 2013-2014 | 2,073 | 2.0 (1.1, 3.7) | 1.0 (0.50, 2.2) |
| Uganda^4^ | | 2011 | 1,187 | 0.84 (0.10, 6.9) | 0.75 (0.12, 4.8) |
| Yemen | | 2013 | 8,476 | 1.4 (0.92, 2.2) | 0.37 (0.23, 0.60) |
| Zambia | | 2013-2014 | 7,520 | 1.2 (0.87, 1.6) | 0.41 (0.29, 0.59) |
| Zimbabwe^4^ | | 2010-2011 | 3,388 | 1.9 (1.2, 2.9) | 0.53 (0.17, 1.7) |
| **Total N/**  **Pooled Odds Ratio** | |  | **162,768** | **1.4 (1.2, 1.7)** | **0.68 (0.58, 0.80)** |

DRC = Democratic Republic of Congo

^1^ Country-specific odds ratios were estimated using logistic regression. Obesity was defined as BMI ≥ 30 kg/m^2^. Country-specific sample sizes are unweighted. Pooled odds ratios were generated using meta-analysis.

^2^ All models were adjusted for maternal age (years), parity, marital status (married, not married), number of household members, child age (months), and substitute childcare provider (yes, no). Models which did not retain the employmentXeducation interaction term were also adjusted for maternal education (< primary education complete, ≥ primary education complete).

^3^ Type of employment was based on 4 indicators: 1) employment during the last 12 months (yes, no); 2) aggregate occupation category (skilled, unskilled); 3) type of earnings (cash only, cash and in-kind, in-kind only, unpaid); and 4) seasonality of employment (all year, seasonal/occasional).

^4^ Included employmentXeducation interaction term.

^5^ Employment type was based on employment status, occupation and earnings only because seasonality of employment was not queried in this survey.

^6^ Maternal education level was dichotomized as < secondary level of education complete and ≥ secondary level of education complete.

^7^ Employment type was based on employment status, type of earnings, and seasonality only because occupation type was not queried in this survey.

| **Table S6. Adjusted linear regression for the relationship between formal and informal maternal employment and BMI^1,2^** | | | | | |
| --- | --- | --- | --- | --- | --- |
|  | |  |  | **β (95% Confidence Interval)** | |
| **Country** | **Year** | **N** | **Formal**  **Employment^3^** | **Informal Employment^3^** |  |
| Bangladesh^4^ | | 2011 | 6,224 | 0.15 (-0.33, 0.64) | -0.012 (-0.72, 0.70) |
| Benin^4^ | | 2011-2012 | 6,372 | 0.47 (-0.020, 0.97) | -0.39 (-0.84, 0.068) |
| Burkina Faso^4^ | | 2010 | 4,204 | 1.1 (0.55, 1.6) | -0.40 (-0.74, -0.065) |
| Cambodia | | 2014 | 3,477 | 0.062 (-0.34, 0.46) | -0.85 (-1.3, -0.45) |
| Cameroon | | 2011 | 2,965 | 0.88 (0.39, 1.4) | 0.00091 (-0.49, 0.49) |
| Colombia^5^ | | 2010 | 11,809 | 0.29 (0.04, 0.54) | 0.39 (0.12, 0.65) |
| Comoros | | 2012 | 1,468 | 0.11 (-0.75, 0.98) | 0.65 (-0.36, 1.7) |
| Cote d'Ivoire | | 2011-2012 | 2,017 | 0.63 (0.021, 1.2) | -0.25 (-0.85, 0.36) |
| DRC^4^ | | 2013-2014 | 4,293 | 0.14 (-0.55, 0.82) | -0.38 (-0.92, 0.15) |
| Dominican Republic | | 2013 | 2,385 | 0.096 (0.53, 0.72) | -0.35 (-1.4, 0.74) |
| Egypt | | 2014 | 9,765 | -0.43 (-0.77, -0.087) | -0.57 (-1.3, 0.14) |
| Ethiopia^4^ | | 2011 | 6,095 | 0.43 (0.015, 0.85) | -0.14 (-0.34, 0.056) |
| Ghana | | 2014 | 1,760 | 0.23 (-0.80, 1.3) | -0.76 (-1.8, 0.27) |
| Guinea | | 2012 | 1,978 | 1.5 (0.86, 2.1) | -0.21 (-0.72, 0.29) |
| Haiti | | 2012 | 2,879 | 0.73 (0.22, 1.2) | 0.010 (-0.47, 0.49) |
| Honduras^4^ | | 2011-2012 | 7,368 | 1.1 (0.43, 1.8) | -0.58 (-1.1, -0.033) |
| Kyrgyz Republic^4,6^ | | 2012 | 2,686 | 2.0 (-0.047, 4.1) | -0.55 (-3.0, 1.9) |
| Lesotho | | 2014 | 1,034 | 0.72 (-0.34, 1.8) | -0.35 (-1.3, 0.57) |
| Liberia | | 2013 | 2,110 | 0.68 (-0.24, 1.6) | -0.37 (-0.87, 0.14) |
| Malawi | | 2010 | 3,026 | 1.4 (0.61, 2.1) | -0.15 (-0.50, 0.20) |
| Mali | | 2012-2013 | 2,308 | 1.7 (0.94, 2.5) | -0.10 (-0.47, 0.27) |
| Mozambique^4^ | | 2011 | 5,848 | 1.9 (1.4, 2.5) | -0.27 (-0.46, -0.066) |
| Namibia | | 2013 | 1,376 | 1.3 (0.64, 2.0) | -0.26 (-1.3, 0.82) |
| Nepal | | 2011 | 1,681 | 0.39 (-0.28, 1.1) | -0.73 (-1.1, -0.32) |
| Niger | | 2012 | 2,771 | 1.3 (0.69, 1.8) | 0.035 (-0.44, 0.51) |
| Nigeria^4^ | | 2013 | 15,052 | 0.25 (-0.079, 0.59) | 0.038 (-0.34, 0.42) |
| Pakistan | | 2012-2013 | 2,106 | -1.1 (-2.1, -0.16) | -2.4 (-3.2, -1.7) |
| Peru^4,7^ | | 2012 | 7,202 | 0.15 (-0.54, 0.85) | -1.8 (-2.5, -1.2) |
| Rwanda^4^ | | 2010 | 2,721 | 1.2 (0.52, 1.9) | 0.13 (-0.29, 0.55) |
| Sierra Leone | | 2013 | 3,194 | 0.51 (-0.12, 1.2) | -0.81 (-1.3, -0.28) |
| Tajikistan | | 2012 | 2,957 | 0.20 (-0.30, 0.69) | -0.57 (-1.0, -0.13) |
| Tanzania^4^ | | 2010 | 4,269 | 2.3 (1.4, 3.1) | 0.31 (-0.28, 0.89) |
| Timor-Leste | | 2009-2010 | 4,724 | 0.42 (0.023, 0.81) | -0.61 (-0.84, -0.37) |
| Togo | | 2013-2014 | 2,073 | 1.0 (0.44, 1.6) | -0.54 (-1.2, 0.14) |
| Uganda^4^ | | 2011 | 1,187 | -0.33 (-1.1, 0.50) | -0.67 (-1.4, 0.092) |
| Yemen | | 2013 | 8,476 | 1.2 (0.47, 1.9) | -0.81 (-1.3, -0.36) |
| Zambia | | 2013-2014 | 7,520 | 0.70 (0.36, 1.1) | -0.87 (-1.1, -0.64) |
| Zimbabwe^4^ | | 2010-2011 | 3,388 | -1.2 (-2.5, 0.0050) | -0.50 (-1.4, 0.42) |
| **Total N/Pooled β** | |  | **162,768** | **0.62 (0.42, 0.82)** | **-0.39 (-0.55, -0.24)** |

BMI = Body Mass Index; DRC = Democratic Republic of Congo

^1^ Beta coefficients were estimated using linear regression to test the relationship between maternal employment and BMI (kg/m^2^). Country-specific sample sizes are unweighted. Pooled coefficients were generated using meta-analysis.

^2^ All models were adjusted for maternal age (years), parity, marital status (married, not married), number of household members, child age (months), and substitute childcare provider (yes, no). Models which did not retain the employmentXeducation interaction term were also adjusted for maternal education (< primary education complete, ≥ primary education complete).

^3^ Type of employment was based on 4 indicators: 1) employment during the last 12 months (yes, no); 2) aggregate occupation category (skilled, unskilled); 3) type of earnings (cash only, cash and in-kind, in-kind only, unpaid), and 4) seasonality of employment (all year, seasonal/occasional).

^4^ Included employmentXeducation interaction term.

^5^ Employment type was based on employment status, occupation and earnings only because seasonality of employment was not queried in this survey.

^6^ Maternal education level was dichotomized as < secondary level of education complete and ≥ secondary level of education complete.

^7^ Employment type was based on employment status, type of earnings, and seasonality only because occupation type was not queried in this survey.

| **Table S7. Adjusted logistic regression for the relationship between formal and informal employment and overweight, among all women^1,2^** | | | | | |
| --- | --- | --- | --- | --- | --- |
|  | |  |  | **Odds Ratio (95% Confidence Interval)** | |
| **Country** | **Year** | **N** | **Formal**  **Employment^3^** | **Informal Employment^3^** |  |
| Bangladesh^4^ | | 2011 | 15,549 | 1.0 (0.78, 1.3) | 0.64 (0.40, 1.0) |
| Benin^4^ | | 2011-2012 | 11,190 | 1.5 (1.3, 1.8) | 0.86 (0.72, 1.0) |
| Burkina Faso^4^ | | 2010 | 6,406 | 1.4 (1.1, 2.0) | 0.44 (0.33, 0.58) |
| Cambodia | | 2014 | 9,575 | 1.0 (0.81, 1.2) | 0.56 (0.45, 0.70) |
| Cameroon | | 2011 | 5,958 | 1.3 (1.1, 1.6) | 0.83 (0.69, 0.99) |
| Colombia^5^ | | 2010 | 38,358 | 1.0 (0.96, 1.1) | 1.2 (1.1, 1.2) |
| Comoros | | 2012 | 3,671 | 1.3 (0.99, 1.6) | 1.0 (0.78, 1.4) |
| Cote d'Ivoire | | 2011-2012 | 3,615 | 1.6 (1.2, 2.0) | 0.86 (0.66, 1.1) |
| DRC^4^ | | 2013-2014 | 6,886 | 1.0 (0.60, 1.8) | 0.67 (0.44, 1.0) |
| Dominican Republic | | 2013 | 7,560 | 1.2 (1.0, 1.4) | 1.2 (0.93, 1.5) |
| Egypt | | 2014 | 19,182 | 1.1 (0.90, 1.3) | 0.81 (0.63, 1.0) |
| Ethiopia^4^ | | 2011 | 12,285 | 2.1 (1.5, 2.9) | 0.51 (0.36, 0.73) |
| Ghana | | 2014 | 3,702 | 1.5 (1.2, 1.9) | 0.92 (0.70, 1.2) |
| Guinea | | 2012 | 3,505 | 1.4 (1.1, 1.8) | 0.53 (0.40, 0.69) |
| Haiti | | 2012 | 7,450 | 1.5 (1.2, 1.8) | 1.1 (0.89, 1.3) |
| Honduras^4^ | | 2011-2012 | 17,922 | 1.6 (1.3, 1.9) | 0.92 (0.77, 1.1) |
| Kyrgyz Republic^4,6^ | | 2012 | 6,416 | 2.0 (1.1, 3.6) | 0.77 (0.25, 2.4) |
| Lesotho | | 2014 | 2,624 | 1.5 (1.2, 1.9) | 1.1 (0.83, 1.4) |
| Liberia | | 2013 | 3,611 | 1.2 (0.83, 1.6) | 0.66 (0.51, 0.87) |
| Malawi | | 2010 | 5,689 | 1.8 (1.4, 2.3) | 1.0 (0.81, 1.3) |
| Mali | | 2012-2013 | 3,502 | 2.1 (1.6, 2.9) | 0.80 (0.63, 1.0) |
| Mozambique^4^ | | 2011 | 10,325 | 3.4 (2.7, 4.2) | 0.74 (0.61, 0.89) |
| Namibia | | 2013 | 3,537 | 2.1 (1.7, 2.5) | 1.2 (0.89, 1.7) |
| Nepal | | 2011 | 4,895 | 0.96 (0.73, 1.3) | 0.34 (0.25, 0.45) |
| Niger | | 2012 | 3,898 | 1.7 (1.3, 2.3) | 1.1 (0.84, 1.4) |
| Nigeria^4^ | | 2013 | 28,698 | 1.4 (1.2, 1.6) | 1.0 (0.82, 1.2) |
| Pakistan | | 2012-2013 | 4,079 | 0.61 (0.47, 0.78) | 0.41 (0.30, 0.57) |
| Peru^4,7^ | | 2012 | 20,009 | 1.1 (0.82, 1.4) | 0.50 (0.39, 0.65) |
| Rwanda^4^ | | 2010 | 5,460 | 1.8 (1.3, 2.7) | 1.1 (0.83, 1.6) |
| Sierra Leone | | 2013 | 6,084 | 1.0 (0.82, 1.3) | 0.52 (0.42, 0.65) |
| Tajikistan | | 2012 | 7,620 | 0.99 (0.84, 1.2) | 0.77 (0.64, 0.93) |
| Tanzania^4^ | | 2010 | 7,683 | 3.9 (2.3, 6.5) | 1.0 (0.65, 1.6) |
| Timor-Leste | | 2009-2010 | 9,293 | 1.1 (0.74, 1.5) | 0.45 (0.34, 0.58) |
| Togo | | 2013-2014 | 3,800 | 1.7 (1.4, 2.2) | 0.64 (0.47, 0.88) |
| Uganda^4^ | | 2011 | 2,014 | 0.91 (0.51, 1.6) | 0.92 (0.54, 1.6) |
| Yemen | | 2013 | 13,838 | 1.5 (1.2, 1.8) | 0.61 (0.49, 0.76) |
| Zambia | | 2013-2014 | 12,455 | 1.5 (1.3, 1.7) | 0.63 (0.56, 0.72) |
| Zimbabwe^4^ | | 2010-2011 | 6,903 | 1.2 (0.67, 2.0) | 1.0 (0.73, 1.4) |
| **Total N/**  **Pooled Odds Ratio** | |  | **345,247** | **1.4 (1.3, 1.5)** | **0.76 (0.68, 0.85)** |

DRC = Democratic Republic of Congo

^1^ Country-specific odds ratios were estimated using logistic regression. Overweight was defined as BMI ≥ 25 kg/m^2^. Country-specific sample sizes are unweighted. Pooled odds ratios were generated using meta-analysis.

^2^ All models were adjusted for maternal age (years), parity, marital status (married, not married), number of household members, child under age two (yes, no), and substitute childcare provider (yes, no). Models which did not retain the employmentXeducation interaction term were also adjusted for maternal education (< primary education complete, ≥ primary education complete).

^3^ Type of employment was based on 4 indicators: 1) employment during the last 12 months (yes, no); 2) aggregate occupation category (skilled, unskilled); 3) type of earnings (cash only, cash and in-kind, in-kind only, unpaid); and 4) seasonality of employment (all year, seasonal/occasional).

^4^ Included employmentXeducation interaction term.

^5^ Employment type was based on employment status, occupation and earnings only because seasonality of employment was not queried in this survey.

^6^ Maternal education level was dichotomized as < secondary level of education complete and ≥ secondary level of education complete.

^7^ Employment type was based on employment status, type of earnings, and seasonality only because occupation type was not queried in this survey.

| **Table S8. Adjusted logistic regression for the relationship between formal and informal employment and overweight, controlling for urban/rural status^1,2^** | | | | | |
| --- | --- | --- | --- | --- | --- |
|  | |  |  | **Odds Ratio (95% Confidence Interval)** | |
| **Country** | **Year** | **N** | **Formal**  **Employment^3^** | **Informal Employment^3^** |  |
| Bangladesh^4^ | | 2011 | 6,224 | 0.87 (0.47, 1.6) | 1.2 (0.48, 3.0) |
| Benin^4^ | | 2011-2012 | 6,372 | 1.3 (1.1, 1.6) | 0.87 (0.70, 1.1) |
| Burkina Faso^4^ | | 2010 | 4,204 | 1.2 (0.80, 1.9) | 0.51 (0.36, 0.74) |
| Cambodia | | 2014 | 3,477 | 1.1 (0.79, 1.4) | 0.54 (0.39, 0.76) |
| Cameroon | | 2011 | 2,965 | 1.2 (0.95, 1.2) | 1.0 (0.77, 1.3) |
| Colombia^5^ | | 2010 | 11,809 | 1.1 (0.97, 1.2) | 1.2 (1.1, 1.40) |
| Comoros | | 2012 | 1,468 | 1.2 (0.81, 1.7) | 1.4 (0.93, 2.1) |
| Cote d'Ivoire | | 2011-2012 | 2,017 | 1.3 (0.91, 1.9) | 0.94 (0.64, 1.4) |
| DRC^4^ | | 2013-2014 | 4,293 | 0.65 (0.34, 1.2) | 0.68 (0.40, 1.2) |
| Dominican Republic | | 2013 | 2,385 | 1.1 (0.83, 1.3) | 0.85 (0.55, 1.3) |
| Egypt | | 2014 | 9,765 | 0.94 (0.75, 1.2) | 0.69 (0.50, 0.97) |
| Ethiopia^4^ | | 2011 | 6,095 | 1.3 (0.70, 2.4) | 0.71 (0.43, 1.2) |
| Ghana | | 2014 | 1,760 | 1.3 (0.91, 2.0) | 1.1 (0.73, 1.7) |
| Guinea | | 2012 | 1,978 | 1.4 (0.92, 2.2) | 0.82 (0.55, 1.2) |
| Haiti | | 2012 | 2,879 | 1.4 (1.1, 1.9) | 1.2 (0.89, 1.6) |
| Honduras^4^ | | 2011-2012 | 7,368 | 1.4 (1.1, 1.9) | 0.86 (0.68, 1.1) |
| Kyrgyz Republic^4,6^ | | 2012 | 2,686 | 3.0 (0.99, 8.9) | 0.51 (0.059, 4.5) |
| Lesotho | | 2014 | 1,034 | 0.97 (0.65, 1.4) | 0.89 (0.59, 1.3) |
| Liberia | | 2013 | 2,110 | 0.90 (0.56, 1.6) | 0.68 (0.62, 1.1) |
| Malawi | | 2010 | 3,026 | 1.7 (1.1, 2.5) | 0.96 (0.70, 1.3) |
| Mali | | 2012-2013 | 2,308 | 1.8 (1.2, 2.7) | 0.83 (0.62, 1.1) |
| Mozambique^4^ | | 2011 | 5,848 | 2.6 (1.8, 3.6) | 0.75 (0.57, 0.98) |
| Namibia | | 2013 | 1,376 | 1.6 (1.1, 2.3) | 0.83 (0.52, 1.3) |
| Nepal | | 2011 | 1,681 | 0.86 (0.49, 1.5) | 0.30 (0.18, 0.51) |
| Niger | | 2012 | 2,771 | 1.5 (1.1, 2.1) | 1.0 (0.72, 1.5) |
| Nigeria^4^ | | 2013 | 15,052 | 1.2 (0.97, 1.5) | 0.93 (0.72, 1.2) |
| Pakistan | | 2012-2013 | 2,106 | 0.52 (0.34, 0.81) | 0.44 (0.28, 0.70) |
| Peru^4,7^ | | 2012 | 7,202 | 0.97 (0.66, 1.4) | 0.55 (0.40, 0.77) |
| Rwanda^4^ | | 2010 | 2,721 | 2.0 (1.1, 3.5) | 1.3 (0.80, 2.0) |
| Sierra Leone | | 2013 | 3,194 | 0.99 (0.72, 1.4) | 0.55 (0.39, 0.78) |
| Tajikistan | | 2012 | 2,957 | 1.0 (0.78, 1.3) | 0.86 (0.65, 1.2) |
| Tanzania^4^ | | 2010 | 4,269 | 3.9 (1.7, 9.1) | 1.6 (0.82, 3.3) |
| Timor-Leste | | 2009-2010 | 4,724 | 0.88 (0.52, 1.5) | 0.51 (0.35, 0.73) |
| Togo | | 2013-2014 | 2,073 | 1.2 (0.88, 1.8) | 0.71 (0.46, 1.1) |
| Uganda^4^ | | 2011 | 1,187 | 0.54 (0.26, 1.1) | 0.62 (0.33, 1.2) |
| Yemen | | 2013 | 8,476 | 1.3 (1.0, 1.7) | 0.70 (0.51, 0.95) |
| Zambia | | 2013-2014 | 7,520 | 1.1 (0.94, 1.4) | 0.73 (0.94, 1.3) |
| Zimbabwe^4^ | | 2010-2011 | 3,388 | 0.47 (0.14, 1.5) | 0.95 (0.58, 1.6) |
| **Total N/**  **Pooled Odds Ratio** | |  | **162,768** | **1.2 (1.1, 1.3)** | **0.80 (0.72, 0.88)** |

DRC = Democratic Republic of Congo

^1^ Country-specific odds ratios were estimated using logistic regression. Overweight was defined as BMI ≥ 25 kg/m^2^. Country-specific sample sizes are unweighted. Pooled odds ratios were generated using meta-analysis.

^2^ All models were adjusted for maternal age (years), parity, marital status (married, not married), number of household members, child under age two (yes, no), substitute childcare provider (yes, no), and within country place of residence (urban, rural). Models which did not retain the employmentXeducation interaction term were also adjusted for maternal education (< primary education, ≥ primary education completed).

^3^ Type of employment was based on 4 indicators: 1) employment during the last 12 months (yes, no); 2) aggregate occupation category (skilled, unskilled); 3) type of earnings (cash only, cash and in-kind, in-kind only, unpaid); and 4) seasonality of employment (all year, seasonal/occasional).

^4^ Included employmentXeducation interaction term.

^5^ Employment type was based on employment status, occupation and earnings only because seasonality of employment was not queried in this survey.

^6^ Maternal education level was dichotomized as < secondary level of education complete and ≥ secondary level of education complete.

^7^ Employment type was based on employment status, type of earnings, and seasonality only because occupation type was not queried in this survey.

| Table S9: The difference in the predicted probability of overweight for formal employment versus non-employment and informal employment versus non-employment | | |
| --- | --- | --- |
|  | **N =162,768^1,2^** | |
|  | **Predicted Probability**  **(95% Confidence Interval)** | |
| Formal Employment^3^ | |  |
| All low- and middle-income countries | | 0.043 (0.026, 0.060) |
| Countries where the association did not vary by education | | 0.040 (0.021, 0.060) |
| Countries where the association varied by education: low education^4^ | | 0.048 (0.016, 0.080) |
| Countries where the association varied by education: high education^4^ | | 0.029 (0.0010, 0.057) |
| Informal Employment^3^ | |  |
| All low- and middle-income countries | | -0.045 (-0.064, -0.026) |
| Countries where the association did not vary by education | | -0.049 (-0.072, -0.026) |
| Countries where the association varied by education: low education^4^ | | -0.037 (-0.070, -0.0040) |
| Countries where the association varied by education: high education^4^ | | -0.076 (-0.11, -0.042) |

^1^ Pooled estimates were generated using meta-analysis and pool estimates across country subgroups. Overweight was defined as BMI ≥ 25 kg/m^2^. All models were adjusted for maternal age (years), parity, marital status (married, not married), number of household members, child age (months), and substitute childcare provider (yes, no). Models which did not retain the employmentXeducation interaction term were also adjusted for maternal education (< primary education, ≥ primary education completed).

^2^ Total sample size is unweighted.

^3^ Type of employment was based on 4 indicators: 1) employment during the last 12 months (yes, no); 2) aggregate occupation category (skilled, unskilled); 3) type of earnings (cash only, cash and in-kind, in-kind only, unpaid); and 4) seasonality of employment (all year, seasonally/occasionally).

^4^ The employmentXeducation interaction term was retained in the following countries: Bangladesh, Benin, Burkina Faso, Democratic Republic of Congo, Ethiopia, Honduras, Kyrgyz Republic, Mozambique, Nigeria, Peru, Rwanda, Tanzania, Uganda, Zimbabwe.

Figure S1. Trends in the maternal employment-overweight associations by log gross domestic product, per capita^1^


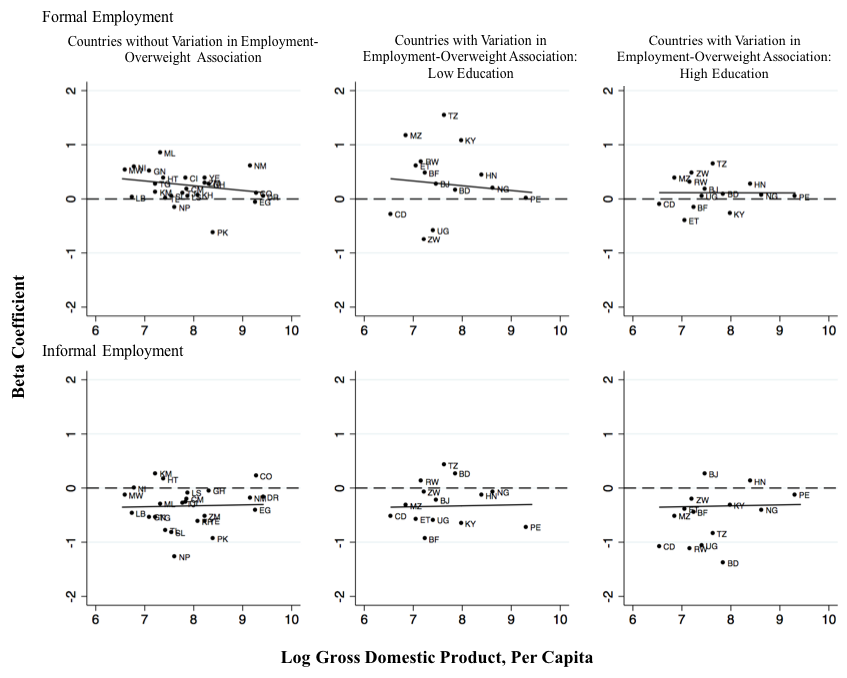


^1^ Data are in international dollars and were obtained from the World Development Indicators database.

Figure S2. Trends in the maternal employment-overweight associations by percent urban population^1^


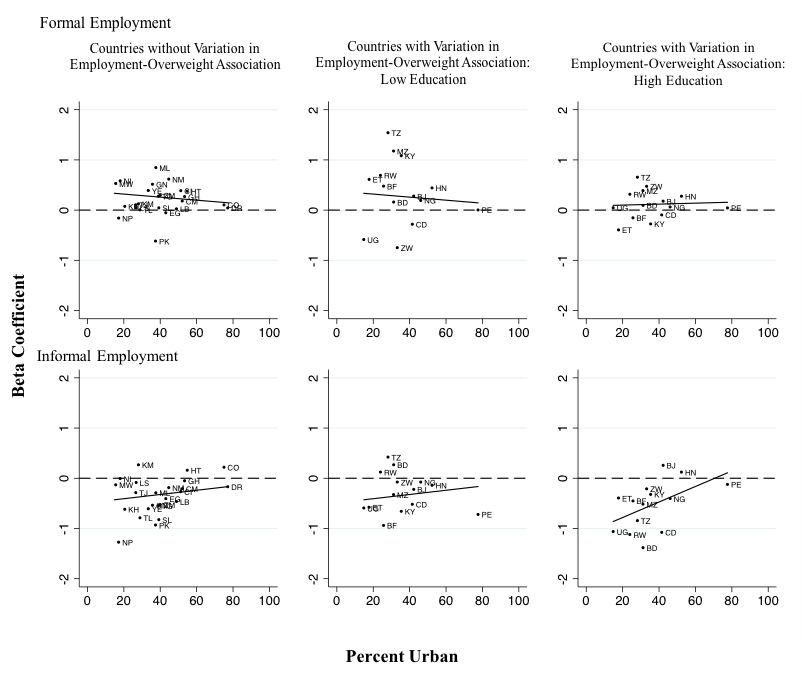


^1^ Urban population refers to people living in urban areas as defined by national statistical offices. The data were obtained from the World Development Indicators database.


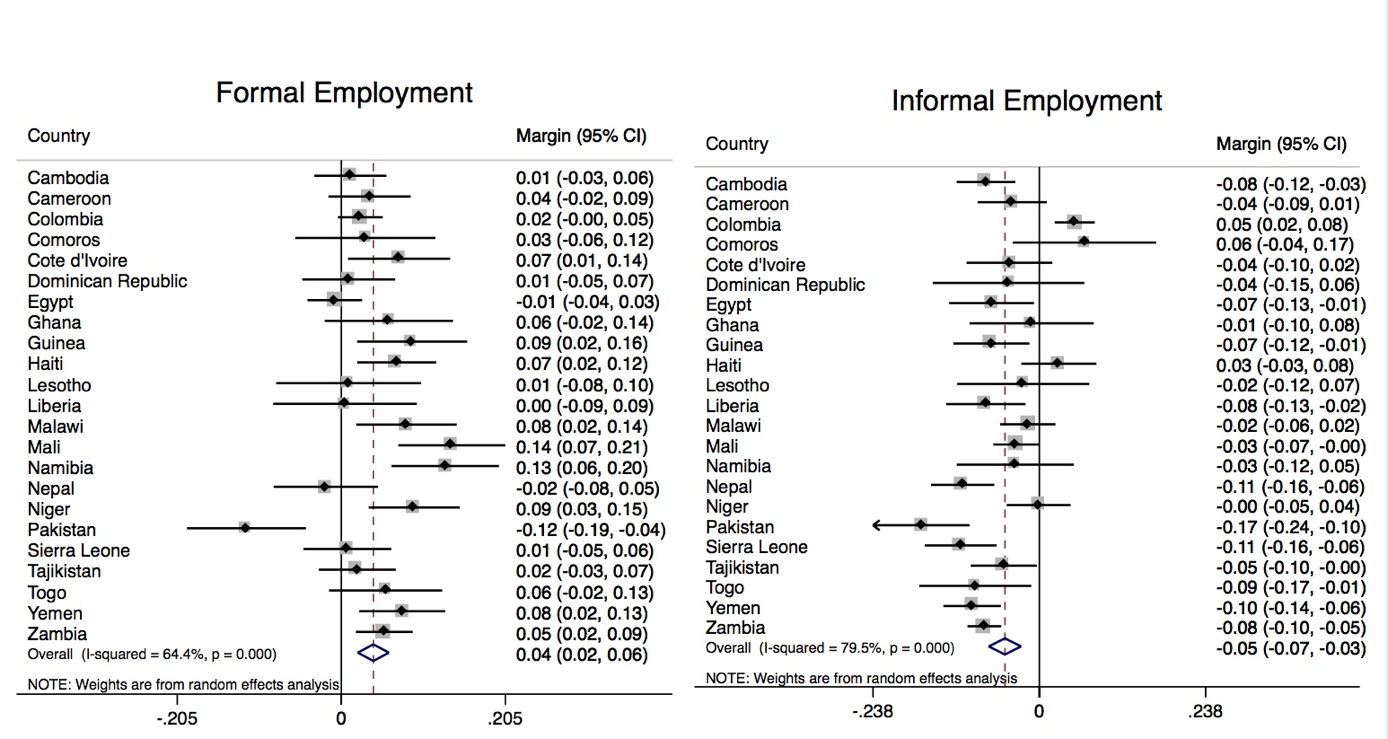
Figure S3. Adjusted difference in the predicted probability of overweight for formal employment versus non-employment and informal employment versus non-employment, in countries where the association did not vary by education^1,2^

^1^ Overall estimates were generated using meta-analysis and pool estimates across countries.

^2^ Models were adjusted for maternal age (years), parity, marital status (married, not married), number of household members, child age (months), substitute childcare provider (yes, no) and maternal education (< primary education, ≥ primary education).

Figure S4. Adjusted difference in the predicted probability of overweight for formal employment versus non-employment and informal employment versus non-employment, in countries where the association did vary by education^1,2^


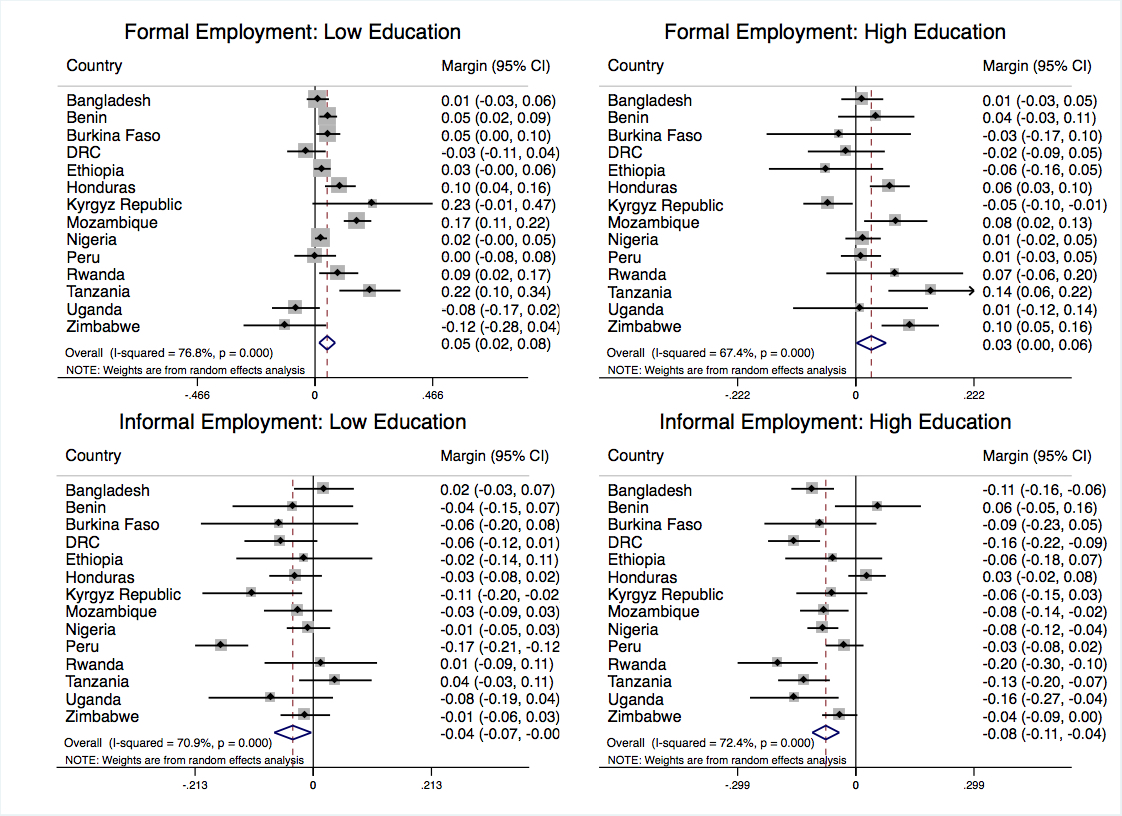


DRC = Democratic Republic of Congo

^1^ Overall estimates were generated using meta-analysis and pool estimates across countries.

^2^ Models were adjusted for maternal age (years), parity, marital status (married, not married), number of household members, child age (months), and substitute childcare provider (yes, no).
